# Editors’ Note

**DOI:** 10.5195/ijt.2016.6209

**Published:** 2016-12-15

**Authors:** ELLEN R. COHN, JANA CASON

The Fall 2016 issue of the International Journal of Telerehabilitation (IJT) presents the original work of a total of 40 authors from six countries: Australia, Cyprus, Latvia, Norway, Greece, and the United States. In contrast to this wide geographic expanse, this issue has a relatively narrow content focus. Much of the content applies to telerehabilitation in older adults – especially persons with chronic obstructive pulmonary disease. This content focus was not intentional. It instead represents the work gratefully received within the past six months, and perhaps the growing salience of these topics.

The current issue presents original and innovative work in four sections: Commentary, Research, Case Studies, and Policy. IJT also publishes clinical reports, book reviews, country reports, and pedagogical notes.

Periodically, IJT features highly integrative commentaries with the potential to be transformational and thought-provoking. This issue’s commentary by authors Marzano, Lubinka, and Stafeckis presents a rationale for Social Telerehabilitation as a sub-category of Telerehabilitation. The authors describe a theoretical model they are developing to facilitate older adults’ access to Information and Communication Technology (ICT) based services. Marzano, Lubinka, and Stafeckis assert the need for “respecting the human personality as a whole (mind, feelings, will, attitude, values, motivation, self-experience, specific features of age, etc.).” They further question whether ICT-services are sufficiently focused on the consumer, versus on the needs of care-takers, observing “…in most cases patients merely appear as input data for computer-based systems, health-care professionals, and policy-makers. There are comparatively fewer ICT-based systems and applications designed to directly benefit older adults.”

The contributors to this and prior IJT issues confirm an intensified global interest in telerehabilitation. Many of the authors who entrust their work to IJT are well-versed in more than one discipline, engage in scholarship in several languages, and move seamlessly between cultures. They are thus able to function well beyond their professional and geographic boundaries. The labels multi-disciplinary, multi-lingual, and multi-cultural do not adequately capture their levels of attainment and sophistication. We are gratified to publish their work.

## CALL FOR SUBMISSIONS

The next volume of the International Journal of Telerehabilitation will be published in Spring, 2017. We cordially invite your submissions by March 8, 2017. IJT accepts original research, case studies, viewpoints, technology reviews, book reviews, and country reports that detail the current status of telerehabilitation. Our peer reviewers constitute a multi-disciplinary group, and include researchers and clinicians from each of the major rehabilitation disciplines, rehabilitation engineers, health information managers, information technologists, and others. We welcome new peer-reviewers and invite guest editors with ideas for special, thematically focused issues. The IJT publication team is agile and can add additional issues as warranted to ensure currency. Please contact Editor Ellen Cohn, PhD (ecohn@pitt.edu) or Senior Associate Editor Jana Cason (jcason@spalding.edu) if you are interested in contributing to a future issue.

Sincerely,

Ellen R. Cohn, PhD, CCC-SLP, ASHA-F, IJT Editor

Jana Cason, DHS, OTR/L, FAOTA, Senior Associate Editor, and Issue Co-Editor

